# Sleep habits and sleep problems among Palestinian students

**DOI:** 10.1186/1753-2000-5-25

**Published:** 2011-07-15

**Authors:** Waleed M Sweileh, Iyad A Ali, Ansam F Sawalha, Adham S Abu-Taha, Sa'ed H Zyoud, Samah W Al-Jabi

**Affiliations:** 1 Department of Pharmacology, College of Pharmacy, An-Najah National University, Nablus, P.O.Box 7, Palestine; 2 Department of Biochemistry, School of Medicine, An-Najah National University, Nablus, P.O.Box 7, Palestine

## Abstract

**Aim:**

The aim of this study was to describe sleep habits and sleep problems in a population of undergraduates in Palestine. Association between self-reported sleep quality and self-reported academic achievement was also investigated.

**Methods:**

Sleep habits and problems were investigated using a convenience sample of students from An-Najah National University, Palestine. The study was carried out during spring semester, 2009. A self-administered questionnaire developed based on The Diagnostic and Statistical Manual of Mental Disorders IV criteria and Pittsburgh Sleep Quality Index was used.

**Results:**

400 students with a mean age of 20.2 ± 1.3 were studied. Reported mean duration of night sleep in the study sample was 6.4 ± 1.1 hours. The majority (58.3%) of students went to bed before midnight and 18% of the total sample woke up before 6 am. Sleep latency of more than one hour was present in 19.3% of the students. Two thirds (64.8%) of the students reported having at least one nocturnal awakening per night. Nightmares were the most common parasomnia reported by students. Daytime naps were common and reported in 74.5% of the study sample. Sleep quality was reported as "poor" in only 9.8% and was significantly associated with sleep latency, frequency of nocturnal awakenings, time of going to bed, nightmares but not with academic achievement.

**Conclusion:**

Sleep habits among Palestinian undergraduates were comparable to those reported in European studies. Sleep problems were common and there was no significant association between sleep quality and academic achievement.

## Background

Prevalence of sleep problems vary based on ethnic and cultural beliefs [1-7]. For example, a study examined differences in sleep complaints among adolescents from nine ethno-cultural groups found that European and American youths were significantly at higher risk of insomnia compared to Chinese Americans after adjusting for age, sex and socioeconomic status [8]. It is believed that disturbances in sleep are associated with poor social performance and various somatic and psychiatric disorders [9-11].

Sleep disorders among university full-time students who are experiencing high levels of stress because of the demands of academic performance is an important topic for investigation [12-14]. However, little research has focused on this group of individuals. Most studies have focused instead on young children, older adults or on a certain category of patients [15-18]. Today's university students experience great psychological pressure due to the changing career market and increased competition for jobs [19]. Such stress and anxiety can lead to sleep problems. In fact, the quality and quantity of sleep of many students might change after enrollment into a university [20]. Sleep deprivation has been reported to cause deleterious effects on medical students [21-25]. Frequent changes in the sleep-wake schedule was also found to adversely affect sleep and general health, including decreased sleep quality, altered sympathetic activity, increased risk of cardiovascular events, and reduced cognitive performance [26,27].

No previously published studies have assessed sleep pattern and sleep problems in undergraduate population in the Middles East. For the purpose of this study, sleep problems were defined as any difficulty in falling asleep or failure to maintain sleep due to noise, nocturnal eating or snoring. Sleep habits were defined as that behavior pertaining to time to bed, time to rise, drinking coffee at night, duration of night sleep and consumption of sleeping pills. In Palestine, where the study took place, the devastating political and military conflict, future insecurity and poverty are expected to affect sleep quality adversely [28-30]. Such factors do not seem to be present in many countries especially the western countries were most sleep studies have been carried out.

The aim of this study was to describe sleep habits and sleep problems in a population of undergraduates in Palestine. Association between self-reported sleep quality and self-reported academic achievement was also investigated.

## 2. Methodology

### 2.1 Study location and sample

This is a cross-sectional, questionnaire-based, observational study carried out in spring 2009 among undergraduate students enrolled at An-Najah National University/Nablus. Approval of University medical research ethics committee was obtained before the initiation of the study. Students were recruited for the study at three main locations in the campus: college of medicine, college of science/engineering and food court. Confidentiality was assured to all students who were asked to volunteer and none were reimbursed. Students who were willing to participate were given a brief description about the study and its objectives. Verbal consent of the student was necessary for his/her enrollment. Students who were currently using sedative medications or narcotics for any acute or chronic medical condition were excluded from the study. Recruitment and collection of data continued for two weeks. The recruitment and collection process was carried out under the supervision of the authors and the help of 25 previously trained senior medical students.

### 2.1 Study tool: the questionnaire

A questionnaire containing 34 questions divided into 6 sections was developed for this study (see Additional file 1). The students were asked to limit their responses to incidents occurred during the past week. The questionnaire was based on Diagnostic and Statistical Manual of Mental Disorders IV (DSM-IV) criteria and Pittsburgh Sleep Quality Index (PSQI) [31]. The questionnaire was initially pilot-tested on a small sample (20 students) and was modified accordingly. The internal validity of the questionnaire was tested using Cronbach's alpha test which was above 0.8 for all tested questions. The questionnaire includes several different types of questions about sleep habits and sleep problems. The first section contained 9 questions about demographic characteristics: gender, age, college, academic level, place of living, permanent residence, body mass index (BMI), day or night workload. The second section contained 5 questions about sleep habits: when do you go to bed, how many hours of sleep do you usually have, when you do usually wake up, do you usually drink coffee at night, and finally have you ever taken sleeping pills. The third section contained 5 questions about sleep problems: how long it takes you to fall asleep, how many times do you wake up during your sleep and do you snore. Causes of failure to maintain sleep were assessed by the following statements: waking up due to noise at night (NN) and waking up because of nocturnal eating habits (NE). The fourth section contained 5 questions pertaining to parasomnia: sleep talking (ST), sleep walking (SW), bruxism (B), nightmares (NM) and restless leg syndrome (RLS). The fifth section contained 5 questions pertaining to daytime tiredness and sleepiness: feeling tired in the morning (TM), daytime sleepiness (DS), daytime sleepiness during lectures (DSL), daytime sleepiness during free time (DSF), and daytime naps (DN). The sixth section contained 5 general questions: subjective feeling about sleep quality, sleep quality on the night before an exam, academic achievement, subjective feeling about his/her leisure time and living conditions.

### 2.3 Statistical analysis

All data were coded, entered, and then analyzed using the Statistical Package for Social Sciences program (SPSS), version 16. Descriptive results were expressed as frequency, percentage, and mean ± S.D. *P*-values < 0.05 were accepted as statistically significant. Pearson chi square was used to test for significant relationships between categorical variables. Non-parametric Spearman Rank Order was used to test for correlation between continuous and ordinal variables. A difference in means between groups was carried out using independent sample t test.

## Results

A total of 400, out of approximately 3000 students, were recruited. There were 207 (51.8%) males. The mean age of all subjects was 20.2 ± 1.3 years (range 17 - 25 years). Eight (2%) students reported having full daytime job and 4 (1%) reported having full nighttime job. Further details about demographic characteristics are shown in Table 1.

**Table 1:** Demographic characteristics of the study sample.

**No**.	Variable	Statistics*
**1**.	Gender	
	1. Male	207 (51.8%)
	2. Female	193 (48.2%)

**2**.	Age (years)	20.2 ± 1.3

**3**.	College	
	1. Medical	203 (50.8%)
	2. Non-Medical	197 (49.2%)

**4**.	Academic level	
	1. First year	77 (19.3%)
	2. Second year	133 (33.2%)
	3. Third year	90 (22.5%)
	4. Fourth year	100 (25%)

**5**.	Place of living	
	1. Student housing	162 (40.4%)
	2. With parents	238 (59.5%)

**6**.	Permanent residence	
	1. City	194 (48.5%)
	2. Village	206 (51.5%)

**7**.	Body Mass Index (BMI)	
	1. Within normal range	380 (95%)
	2. Above or below normal range	20 (5%)

**8**.	Do you work during day?	
	1. Never	306 (76.5%)
	2. Sometimes	75 (18.8%)
	3. Part-time	11 (2.8%)
	4. Full time	8 (2%)

**9**.	Do you work at night?	
	1. Never	353 (88.2%)
	2. Sometimes	40 (10%)
	3. Part-time	3 (0.8%)
	4. Full time	4 (1%)

*Statistics are expressed as mean ± SD for continuous variables and as a frequency for categorical variables.

### Sleep habits

Analysis of time to go to bed showed that 124 (31%) students went to bed before 10 pm, 109 (27.3%) went to bed between 10 pm and midnight, and 167 (41.7%) went to bed after midnight. The average duration of sleep reported by students was 6.4 ± 1.1 hours. Two hundred and thirty four (58.5%) students reported that they never had coffee late at night while 113 (28.4%) students had coffee late at night at least 1 - 2 per week. Drinking coffee late at night was significantly correlated with time of going to bed (p = 0.001, r = 0.2). Seventy two (18%) students woke up before 6 am while the majority (189, 47.3%) of the students woke up between 6 - 8 am. Finally, only 4 (1%) students reported using medications to enhance their sleep (Table 2).

**Table 2:** Sleep habits of the study subjects.

**No**.	Variable	Statistics*
**1**.	When do you usually go to bed?	
	1. < 10 pm	124 (31%)
	2. 10 - 12 pm	109 (27.3%)
	3. > 12 pm	167 (41.7%)

**2**.	How many hours of sleep do you usually have?	6.4 ± 1.1

**3**.	When do you usually wake up?	
	1. < 6 am	72 (18%)
	2. 6 - 8 am	189 (47.3%)
	3. > 8 am	139 (34.7%)

**4**.	Do you drink coffee late at night?	
	1. Never	234 (58.5%)
	2. < once per week	53 (13.2%)
	3. 1 - 2 per week	43 (10.85)
	4. 3 - 5 per week	27 (6.8%)
	5. nightly	43 (10.8%)

**5**.	Have you ever taken sleeping pills	
	1. < once per week	14 (3.5%)
	2. Never	386 (96.5%)

### Sleep problems

Results of question pertaining to how long it takes the student to fall asleep (sleep latency) showed that 114 (28.6%) students had a sleep latency of < 10 minutes; 141 (35.2%) had a sleep latency of 10 - 30 minutes; 68 (17%) had a sleep latency of 30 - 60 minutes; and 77 (19.2%) had a sleep latency of > 60 minutes (Table 3). The question on the frequency of nocturnal awakenings showed the following results: never in 141 (35.2%); 1 - 2 times per night in 201 (50.2%); 3 - 4 times per night in 46 (11.5%); > 5 times per night in 12 (3%). Only 13 (3.2%) students reported having a snoring problem every night. Night noise (NN) was reported to be the main cause of sleep interruption (Table 4).

**Table 3:** Prevalence of sleep related problems in the studied sample.

**No**.	Variable	Statistics*
**1**.	How long it takes you to actually fall asleep (sleep latency)?	
	1. < 10 minutes	114 (28.6%)
	2. 10 - 30 minutes	141 (35.2%)
	3. 30 - 60 minutes	68 (17%)
	4. > 60 minutes	77 (19.25)

**2**.	How many times do you wake up during your sleep (nocturnal awakenings)?	
	1. None	141 (35.2%)
	2. 1- 2	201 (50.3%)
	3. 3 - 4	46 (11.5%)
	4. > 5	12 (3%)

**3**.	Do you snore	
	1. Never	349 (87.2%)
	2. < once per week	27 (6.8%)
	3. 1 - 2 per week	8 (2%)
	4. 3 - 5 per week	3 (0.8%)
	5. Nightly	13 (3.2%)

**Table 4:** Causes of failure to maintain sleep.

No	Variable	N (%)				
		**Never**	**Less than once a week**	**1 - 2 nights a week**	**3 - 4 nights a week**	**Almost nightly/daily**

1.	NN	158 (39.5%)	90 (22.5%)	61 (15.2%)	23 (5.2%)	68 (17%)

2.	NE	290 (72.5%)	67 (16.8%)	23 (5.8%)	7 (1.8%)	13 (3.25)

### Parasomnia

Parasomnias such as sleep-talking (ST), sleep walking (SW), bruxism (B), nightmares (NM), and rest leg syndrome (RLS) were investigated (Table 5). Sleep walking was the least common while nightmares were the most common type of parasomnia. Spearman correlation test between sleep problems and parasomnia was carried out. No significant correlation was found between sleep latency and any of the parasomnia disorders.

**Table 5:** Prevalence of parasomnia in the study sample.

Type of parasomnia	N (%)				
	**Never**	**Less than once a week**	**1 - 2 nights a week**	**3 - 4 nights a week**	**Almost nightly/daily**

ST	309 (77.2%)	40 (10%)	24 (6%)	5 (1.2%)	22 (5.5%)

SW	377 (94.2%)	14 (3.5%)	2 (0.5%)	3 (0.8%)	4 (1.0%)

B	340 (85%)	26 (6.5%)	18 (4.5%)	7 (1.8%)	9 (2.2%)

NM	214 (53.5%)	111 (27.8%)	51 (12.8%)	10 (2.5%)	14 (3.5%)

RLS	246 (61.5%)	105 (26.2%)	34 (8.5%)	7 (1.8%)	8 (2%)

### Daytime sleepiness and tiredness

Regarding the question about feeling tired in the morning (TM), 78 (19.5%) students never had TM and 220 (55%) had TM at least once a week. Daytime sleepiness (DS) was reported as follows: 63 (15.8%) never had DS while 285 (71.3%) had DS at least once a week. When asked about daytime sleepiness during lectures (DSL) the answers were like follows: 49 (12.2%) never had DSL while 288 (72%) had DSL at least once a week. Regarding daytime sleepiness during free time (DSF), 164 (41%) students never had DSF while 146 (36.5%) had DSF at least once a week. Finally, the question about daytime naps (DN) was answered as follows, 102 (25.5%) never had DN while 232 (58%) had DN at least once a week (Table 6). Feeling tired in the morning was significantly correlated with NM (r = 0.1, p = 0.037), and duration of night sleep (r = 0.1, p = 0.04). Daytime sleepiness was significantly correlated with NM (r = 0.1, p = 0.033), but not with duration of night sleep (p = 0.4).

**Table 6:** Prevalence of daytime sleepiness and tiredness in the study sample.

Variable	N (%)				
	**Never**	**Less than once a week**	**1 - 2 nights a week**	**3 - 4 nights a week**	**Almost nightly/daily**

TM	78 (19.5%)	102 (25.5%)	86 (21.5%)	44 (11%)	90 (22.4%)

DS	63 (15.8%)	52 (13%)	95 (23.8%)	81 (20.2%)	109 (27.2%)

DSL	49 (12.2%)	63 (15.8%)	91 (22.8%)	63 (15.8%)	134 (33.5%)

DSF	164 (41.0%)	90 (22.5%)	67 (16.8%)	30 (7.5%)	49 (12.2%)

DN	102 (25.5%)	66 (16.5%)	92 (23.0%)	67 (16.8%)	73 (18.2%)

### Sleep quality

Upon asking students about sleep quality, the following results were obtained (Table 7): excellent (87, 21.8%); good (201, 50.2%); satisfactory (73, 18.2%); poor (39, 9.8%). Spearman Order Rank test was carried between reported sleep quality and most sleep problems (Table 8). There was a significant positive correlation between reported subjective sleep quality and the following variables: time of going to bed (r = 0.2, P < 0.01); sleep latency (r = 0.23, P < 0.01); frequency of nocturnal awakenings (r = 0.15, p < 0.01); waking up due to noise (r = 0.1, P < 0.04); nightmares (r = 0.1, p < 0.04); feeling tired in the morning (r = 0.25, P < 0.01); daytime sleepiness (r = 0.1, p < 0.01); daytime sleepiness during lectures (r = 0.2, P < 0.01), leisure time (r = 0.2, p < 0.01) and finally living conditions (r = 0.1, p = 0.04). There was no significant correlation between sleep quality and other variable including academic achievement.

**Table 7:** Sleep quality and academic achievement of students in the study sample.

Variable	Excellent	Good	Satisfactory	Poor
sleep Quality	87 (21.8%)	201 (50.25)	73 (18.2%)	39 (9.8%)

Sleep quality on the night before an exam	40 (10%)	103 (25.8%)	91 (22.8%)	166 (41.4%)

Academic achievement	44 (11%)	210 (52.5%)	102 (25.5%)	44 (11%)

Leisure time	33 (8.2%)	168 (42%)	127 (31.8%)	72 (18%)

Living conditions	84 (21%)	219 (54.8%)	71 (17.8%)	26 (6.5%)

**Table 8:** Correlation between sleep quality, academic achievement and sleep habits/problems in the study sample.

	Variable	Sleep Quality		Academic achievement	
		**Correlation Coefficient**	**p**	**Correlation Coefficient**	**p**

1.	Academic level	0.02	0.7	0.01	0.8

2.	Time of going to bed	0.2	**< 0.01**	0.08	0.1

3.	Usual time to wake up	0.06	0.2	0.01	0.8

4.	Duration of sleep	0.08	0.09	0.01	0.8

5.	Drinking coffee late at night	0.01	0.052	0.02	0.07

6.	Use of sleeping pills	0.04	0.5	0.01	0.08

7.	Time to fall asleep (latency)	0.23	**< 0.01**	0.06	0.3

8.	Frequency of nocturnal awakenings	0.15	**< 0.01**	0.03	0.6

9.	Snoring	0.05	0.3	0.01	0.8

10.	Waking up due to nocturnal noise	0.1	**0.02**	0.01	0.08

11.	Waking up because of nocturnal eating	0.04	0.4	0.01	0.8

12.	Sleep talking	0.01	0.9	0.1	0.05

13.	Sleepwalking	0.01	0.9	0.01	0.09

14.	Bruxism	0.01	0.9	0.03	0.5

15.	Nightmares	0.1	**0.046**	0.1	**0.024**

16.	Restless leg syndrome	0.01	0.8	0.06	0.2

17.	Feeling tired in the morning	0.25	**< 0.01**	0.01	0.8

18.	Daytime sleepiness	0.1	**0.016**	0.03	0.5

19.	Daytime sleepiness during lectures	0.2	**< 0.01**	0.07	0.2

20.	Daytime sleepiness during free time	0.01	0.8	0.02	0.7

21.	Daytime naps	0.01	0.8	0.01	0.9

22.	Sleep quality	-	-	0.02	0.7

23.	Academic achievement	0.02	0.7	-	-

24.	Leisure time	0.2	**< 0.01**	0.4	**< 0.01**

25.	Living conditions	0.1	**0.04**	0.24	**< 0.01**

### Academic achievement

Upon asking students about academic achievement on a four point scale, the following results were obtained: excellent (44, 11%); good (210, 52.5%); satisfactory (102, 25.5%); poor (44, 11%). Spearman Order Rank test was carried between academic achievement and most sleep problems (Table 8). There was a significant correlation between academic achievement and nightmares (r = 0.1, p = 0.024), where students with higher nightmare frequency have low academic achievement. On the other hand, leisure time (r = 0.4, p < 0.01) and living conditions (r = 0.24, p < 0.01) were positively correlated with better academic achievement. There was no significant correlation between academic achievement and parasomnia or other sleep problems.

## Discussion

### Sleep habits and sleep problems

This is the first study to describe sleep habits and sleep problems among undergraduates in Palestine and in the Middle East. In our study, approximately 42% went to bed after midnight and 18% woke up before 6 am. Bedtime has been associated with modern lifestyles [32-34]. In Egypt, France, UK, Germany, and Italy bedtime has been shown to be close to midnight [35,36]. Our study showed that the average duration of night sleep among Palestinian undergraduates was 6.4 ± 1.1 hours. A study reported average sleep duration among Korean college students to be 6.7 +/- 1.3 hours [37]. A similar study among Chinese college students reported an average duration of sleep during weekdays to be 6.9 hours [38]. Comparable results were by a study among African college students in Nigeria who reported average sleep duration of 6.2 hours [39]. Studies of general populations and of students populations above 16 years of age [40-43] have shown that the average duration of night sleep is < 8 hours [40-43]. In our study the vast majority of the students never used medication to enhance sleep. This rate is very similar to that reported from other Islamic countries [41]. Our results showed that 36.3% of the participants took more than 30 minutes to fall asleep and 64.8% woke up more than once a night. These data suggest sleep difficulties consistent with research of the National Sleep Foundation as well as studies published among college students in USA which reported that more than 40% of Americans have difficulty falling asleep or have night waking [44,45].

Studies from different countries have shown that many college students are at risk for sleep disorders, and those at risk may also be at risk for academic failure [46] Timing of sleep and wakefulness correlated more closely with academic performance than total sleep time and other relevant factors. These findings have important implications for programs intended to improve academic performance by targeting sleep habits of students [47].

### Daytime tiredness and sleepiness

Our study showed that sleepiness during the day was common Palestinian undergraduates. Most students in our study experienced daytime sleepiness more than half reported having a daytime nap at least once per week. Daytime nap is a popular habit among Arabs in the Middle East [17]. Lower prevalence rate of daytime sleepiness was reported among adults in Europe. In four European countries, 23% of young adults reported having daytime naps [36]. Studies did not show an adverse affect of daytime naps on night sleep [48].

### Parasomnia

In our study, 12.3% of the students experienced RLS at least one night per week. Restless leg syndrome is estimated to occur in 5-15% of the general population [49]. In our study, 8.5% experienced bruxism at least one night per week which is within the range of 6 - 20% reported in the general population [50-52]. However, the incidence of nightmares, sleep talking and sleep walking in this study was greater than that reported in a European study [53]. This can be explained by the political and economical instability in our region. Frequent Israeli military invasion to Palestinian cities during night hours might be a crucial element in the presence of nightmares and sleep quality in general among Palestinian undergraduates.

### Sleep quality and academic achievement

Our study demonstrated that complaints about sleep problems are common among university students. Approximately 28% of students evaluated their sleep quality as satisfactory or poor. Poor sleep quality or sleep deprivation might impair memory and learning process among students [54]. Therefore, it is believed that university students who suffer from sleep disorders have a major risk of poor academic performance compared to those who have had an adequate amount of sleep [53]. Our results showed that reported sleep quality was not associated with academic progress which is not in agreement with other studies [52,55,56].

### Limitations

Sleep problems may be worse than those reported in our study, as students may give socially desirable answers such as not having sleep problems. Thus, this survey may be limited by underreporting. Furthermore, this study reported sleep patterns and problems in the past week which might be representative of the students' general sleep behavior. Other limitations include the fact that the questionnaire was self constructed and no sleep diary was included. This study has imitations that might affect the results obtained. First, other factors affecting sleep such as overcrowded homes, sharing the room with other students, watching TV and internet use were not included in the study. Inclusion of such factors could have resulted in a lengthy questionnaire that could have made students unwilling to participate. Secondly, this study was a cross-sectional study based only on the previous week. Further studies based on longer period with separate data on week days and weekends are needed. Comparison between different studies in different countries is not an easy task because there is much variability in operational definitions and different measures are used to evaluate sleep. For example, in Italy, 18 year-old adolescents are still attending high school and living with their families while in USA they are already attending college.

## Conclusions

In conclusion, this study shows that sleep habits among Palestinian undergraduates were comparable to those reported in European studies. Sleep problems were common and there was no significant association between sleep quality and academic achievement.

## List of abbreviations

B: bruxism; DN: daytime naps; DS: daytime sleepiness; DSF: daytime sleepiness in free time; DSS: daytime sleepiness during classes; NE: waking up because of nocturnal eating habits; NM: nightmares; NN: waking up due to noise at night; RLS: restless leg syndrome; S: snoring; ST: sleep-talking; SW: sleepwalking; TM: feeling tired in the morning.

## Competing interests

The authors declare that they have no competing interests.

## Authors' contributions

All authors read and approved the final manuscript.

WS analyzed the data and wrote the manuscript. IA helped in data analysis and literature review. AS designed the project and reviewed the analysis. AA, SZ and SA collected and entered the data into SPSS.

## Supplementary Material

Additional file 1 **Sleep questionnaire used to evaluate sleep pattern and sleep problems in the study sample**. A sleep questionnaire composed of 35 questions was constructed based on Diagnostic and Statistical Manual of Mental Disorders IV criteria and Pittsburgh Sleep Quality Index. Click here for file

## References

[B1] Bixler EO Kales A Soldatos CR Kales JD Healey S Prevalence of sleep disorders in the Los Angeles metropolitan area Am J Psychiatry 1979 136 1257 1262 314756 10.1176/ajp.136.10.1257

[B2] Quera-Salva MA Orluc A Goldenberg F Guilleminault C Insomnia and use of hypnotics: study of a French population Sleep 1991 14 386 391 1759090 10.1093/sleep/14.5.386

[B3] Hetta J Broman JE Mallon L Evaluation of severe insomnia in the general population--implications for the management of insomnia: insomnia, quality of life, and healthcare consumption in Sweden J Psychopharmacol 1999 13 S35 S36 10667458 10.1177/026988119901304S10

[B4] Hoffmann G Evaluation of severe insomnia in the general population--implications for the management of insomnia: focus on results from Belgium J Psychopharmaco 1999 13 S31 S32 10.1177/026988119901304S08 10667456

[B5] Doi Y Minowa M Okawa M Uchiyama M 2000. Prevalence of sleep disturbance and hypnotic medication use in relation to sociodemographic factors in the general Japanese adult population J Epidemiol 2000 10 79 86 10778031 10.2188/jea.10.79

[B6] Ohayon MM Zulley J Correlates of global sleep dissatisfaction in the German population Sleep 2001 24 780 787 11683481

[B7] Leger D Guilleminault C Dreyfus JP Delahaye C Paillard M Prevalence of insomnia in a survey of 12,778 adults in France J Sleep Res 2000 9 35 42 10.1046/j.1365-2869.2000.00178.x 10733687

[B8] Roberts RE Roberts CR Chen IG Ethnocultural differences in sleep complaints among adolescents J Nerv Ment Dis 2000 188 222 9 10.1097/00005053-200004000-00005 10789999

[B9] Partinen M Guilleminault C Daytime sleepiness and vascular morbidity at seven-year follow-up in obstructive sleep apnea patients Chest 1999 97 27 31 10.1378/chest.97.1.27 2295260

[B10] Newman AB Enright PL Manolio TA Sleep disturbance, psychosocial correlates, and cardiovascular disease in 5201 older adults: the cardiovascular health study J Am Geriatr Soc 1999 45 1 7 10.1111/j.1532-5415.1997.tb00970.x 8994480

[B11] Rocha LF Guerra HL Lima-Costa MFF Prevalence of insomnia and associated socio-demographic factors in a Brazilian community: the Bambui study Sleep Med 2002 3 121 126 10.1016/S1389-9457(01)00119-8 14592230

[B12] Egan KG Moreno MA Prevalence of Stress References on College Freshmen Facebook Profiles Comput Inform Nurs 2011 in press 10.1097/NCN.0b013e3182160663 PMC3205256 21436681

[B13] Lală A Bobîrnac G Tipa R Stress levels, alexithymia, type A and type C personality patterns in undergraduate students J Med Life 2010 3 2 200 5 20968210 PMC3019056

[B14] El-Gilany AH Amr M Hammad S Perceived stress among male medical students in Egypt and Saudi Arabia: effect of sociodemographic factors Ann Saudi Med 2008 28 6 442 8 10.4103/0256-4947.51666 19011321 PMC6074256

[B15] Liu J Hay J Joshi D Faught BE Wade T Cairney J Sleep difficulties and obesity among preadolescents Can J Public Health 2011 102 2 139 43 21608387 10.1007/BF03404163 PMC6973892

[B16] Ikeda T Nagai T Kato-Nishimura K Mohri I Taniike M Sleep problems in physically disabled children and burden on caregivers Brain Dev 2011 in press 10.1016/j.braindev.2011.04.011 21602006

[B17] Wali SO Krayem AB Samman YS Mirdad S Alshimemeri AA Almobaireek A Sleep disorders in Saudi health care workers Ann Saudi Med 1999 19 5 406 9 17277504 10.5144/0256-4947.1999.406

[B18] BaHammam A Bin Saeed A Al-Faris E Shaikh S Sleep duration and its correlates in a sample of Saudi elementary school children Singapore Med J 2006 47 10 875 81 16990963

[B19] Biggeri L Bini L Grilli L The transition from university to work: a multilevel approach to the analysis of the time to obtain the first job 2001 164 2 293 305

[B20] Pilcher JJ Ginter DR Sadowsky B Sleep quality versus sleep quantity: relationships between sleep and measures of health, well-being and sleepiness in college students J Psychosom Res 1997 42 6 583 596 10.1016/S0022-3999(97)00004-4 9226606

[B21] Veasey S Rosen R Barzansky B Rosen I Owens J Sleep loss and fatigue in residency training: a reappraisal J Am Med Assoc 2002 288 1116 1124 10.1001/jama.288.9.1116 12204082

[B22] Fletcher KE Underwood W III Davis SQ Mangrulkar RS McMahon LF Jr Saint S Effects of work hour reduction on residents' lives: a systematic review J Am Med Assoc 2005 294 1088 1100 10.1001/jama.294.9.1088 16145030

[B23] Greenfield LJ Limiting resident duty hours Am J Surg 2003 185 10 12 10.1016/S0002-9610(02)01144-3 12531436

[B24] Parthasarathy S Sleep and the medical profession Curr Opin Pulm Med 2005 11 507 512 16217176 10.1097/01.mcp.0000183060.60547.40

[B25] Landrigan CP Sliding down the bell curve: effects of 24-hr work shifts on physicians's cognition and performance Sleep 2005 28 1351 1353 16335321 10.1093/sleep/28.11.1351

[B26] Owens JA Veasey SC Rosen RC Physician. Heal thyself: sleep, fatigue and medical education Sleep 2001 24 493 495 11480647 10.1093/sleep/24.5.493

[B27] Howard SK Gaba DM Rosekind MR Zarcone VP The risks and implication of excessive daytime sleepiness in resident physicians Acad Med 2002 77 1019 1025 10.1097/00001888-200210000-00015 12377678

[B28] Baker A Shalhoub-Kevorkian N Effects of political and military traumas on children: the Palestinian case Clin Psychol Rev 1999 19 8 935 50 10.1016/S0272-7358(99)00004-5 10547711

[B29] Abu-Mourada T Koutis A Alegakis A Markaki A Jildeh C Lionis C Philalithis A Self-reported health complaints in a primary care population living under stressful conditions in the Gaza Strip, Palestine Med Confl Surviv 2010 26 1 68 79 10.1080/13623690903553269 20411856

[B30] Qouta S Odeb J The impact of conflict on children: the Palestinian experience J Ambul Care Manage 2005 28 1 75 9 15682964 10.1097/00004479-200501000-00010

[B31] Buysse DJ Reynols CF Monk TH Berman SR Kupfer DJ The Pittsburg Sleep Quality Index: a new instrument for psychiatric practice and research Psychiatry Res 1989 28 192 213 10.1016/0165-1781(89)90047-4 2748771

[B32] Carskadon MA Wolfson AR Acebo C Tzischinsky O Seifer R Adolescent sleep patterns, circadian timing, and sleepiness at a transition to early school days Sleep 1998 21 871 81 9871949 10.1093/sleep/21.8.871

[B33] Smedje H Australian study of 10- to 15-year olds shows significant decline in sleep duration between 1985 an 2004 Acta Paediatr 2007 96 954 5 10.1111/j.1651-2227.2007.00351.x 17577337

[B34] Patel SR Social and demographic factors related to sleep duration Sleep 2007 30 1077 8 17910376 10.1093/sleep/30.9.1077 PMC1978396

[B35] Worthman CM Brown RA Companionable sleep: social regulation of sleep and cosleeping in Egyptian families J Fam Psychol 2007 21 124 35 17371117 10.1037/0893-3200.21.1.124 PMC3771325

[B36] Ohayon MM Shapiro CM Sleep and fatigue Semin Clin Neuropsychiatry 2000 5 56 7 10704538 10.153/SCNP00500056

[B37] Ban DJ Lee TJ Sleep duration, subjective sleep disturbances and associated factors among university students in Korea J Korean Med Sci 2001 16 4 475 80 11511794 10.3346/jkms.2001.16.4.475 PMC3054766

[B38] Tsui YY Wing YK A study on the sleep patterns and problems of university business students in Hong Kong J Am Coll Health 2009 58 2 167 76 10.1080/07448480903221418 19892654

[B39] Oluwole OS Sleep habits in Nigerian undergraduates Acta Neurol Scand 2010 121 1 1 6 Epub 2009 Nov 26 19951272 10.1111/j.1600-0404.2009.01171.x

[B40] Groeger JA Zijlstra FR Dijk DJ Sleep quantity, sleep difficulties and their perceived consequences in a representative sample of some 2000 British adults J Sleep Res 2004 13 359 71 10.1111/j.1365-2869.2004.00418.x 15560771

[B41] Ghanizadeh A Kianpoor M Rezaei M Sleep patterns and habits in high school students in Iran. Annals of General Psychiatry 2008 7 http://www.annals-general-psychiatry.com/content/7/1/5. 10.1186/1744-859X-7-5 PMC2292723 18339201

[B42] Joo S Shin C Kim J Prevalence and correlates of excessive daytime sleepiness in high school students in Korea Psychiatry Clin Neurosci 2005 59 433 40 10.1111/j.1440-1819.2005.01396.x 16048449

[B43] Loessl G Valerius G Kopasz M Hornyak M Riemann D Voderholzer U Are adolescents chronically sleep-deprived? An investigation of sleep habits of adolescents in the Southwest of Germany Child Care Health Dev 2008 34 549 56 10.1111/j.1365-2214.2008.00845.x 18549435

[B44] National Sleep Foundation The Basics of Sleep http://www.sleepfoundation.org/Accessed June 17, 2011

[B45] Forquer LM Camden AE Gabriau KM Johnson CM Sleep patterns of college students at a public university J Am Coll Health 2008 56 5 563 5 10.3200/JACH.56.5.563-565 18400669

[B46] Gaultney JF The prevalence of sleep disorders in college students: impact on academic performance J Am Coll Health 2010 59 2 91 7 10.1080/07448481.2010.483708 20864434

[B47] Eliasson AH Lettieri CJ Eliasson AH Early to bed, early to rise! Sleep habits and academic performance in college students Sleep Breath 2010 14 1 71 5 Epub 2009 Jul 15 10.1007/s11325-009-0282-2 19603214

[B48] Dhand UK Dhand R Sleep disorders in neuromuscular diseases Curr Opin Pulm Med 2006 12 402 8 10.1097/01.mcp.0000245704.92311.1e 17053488

[B49] Lavigne GJ Montplaisir JY Restless legs syndrome and sleep bruxism: prevalence and association among Canadians Sleep 1994 17 8 739 43 7701186

[B50] Goulet JP Lund JP Montplaisir JY Daily clenching, nocturnal bruxism, and stress and their association with TMD symptoms J Orofacial Pain 1993 1 120

[B51] Thorpy MJ International classification of sleep disorders: diagnostic and coding manual Diagnostic Classification Steering Committee-American Sleep Disorders Association 1990 Rochester, Minnesota

[B52] Thorpy MJ Yager J The encyclopedia of sleep and sleep disorders New York 2001

[B53] Veldi M Aluoja A Vasar V Sleep quality and more common sleep-related problems in medical students Sleep Med 2005 6 269 75 10.1016/j.sleep.2004.12.003 15854858

[B54] Curico G Ferrara M Gennaro LD 2006. Sleep loss, learning capacity, and academic performance Sleep Med Rev 2006 10 323 337 10.1016/j.smrv.2005.11.001 16564189

[B55] Canellas RL Palmer A Calafat A 1994. Adolescents' sleep characteristic in Mallorca Sleep Res 1994 23 240

[B56] Yarcheski A Mahon NE A study of sleep during adolescence J Pediatr Nurs 1994 7837054

