# Supplementary material for: Sleep habits and sleep problems among Palestinian students
Source: Child Adolesc Psychiatry Ment Health. 2011 Jul 15;5:25. doi: 10.1186/1753-2000-5-25 (PMC3148974; doi:10.1186/1753-2000-5-25)
Supplement: Additional file 1 — Sleep questionnaire used to evaluate sleep pattern and sleep problems in the study sample. A sleep questionnaire composed of 35 questions was constructed based on Diagnostic and Statistical Manual of Mental Disorders IV criteria and Pittsburgh Sleep Quality Index. [file 1753-2000-5-25-S1.DOC]

**Appendix 1**

Questionnaire (questions 10, 11, 12 and 31 are adopted from Pittsburgh Sleep Quality Index (PSQI).

**Demographics**

1. Gender
2. Age
3. College
4. Academic level
5. Place of living
6. Permanent residence
7. Body Mass Index
8. Do you work during day? If yes, how many hours?
9. Do you work at night? If yes, how many hours?

**Sleep habits and Duration of sleep**

1. When do you go to sleep?
2. How many hours of sleep do you usually have?
3. When do you usually wake up?
4. Do you drink coffee late at night (never, <1 per week, 1 – 2 per week, 3 – 4 per week, almost nightly)
5. Do you use sleeping pills to induce sleep?

**Sleep problems**

1. How long it takes you to actually fall asleep? (< 10, 10 – 30, 30 – 60, > 60 minutes)
2. How many times do you wake up during your sleep?
3. Do you snore (never, <1 per week, 1 – 2 per week, 3 – 4 per week, almost nightly)

|  | Answer the following questions based on your sleep behavior in the previous week | **Never** | **Less than once a week** | **1 – 2 nights a week** | **3 – 4 nights a week** | **Nightly/ Daily** |
| --- | --- | --- | --- | --- | --- | --- |
| 18. | Waking up due to noise |  |  |  |  |  |
| 19. | Waking up because of nocturnal eating |  |  |  |  |  |

**Parasomnia**

|  | Answer the following questions based on your sleep behavior in the previous week | **Never** | **Less than once a week** | **1 – 2 nights a week** | **3 – 4 nights a week** | **Nightly/ Daily** |
| --- | --- | --- | --- | --- | --- | --- |
| 22. | Sleep talking |  |  |  |  |  |
| 23. | Sleepwalking |  |  |  |  |  |
|  | Nocturnal bruxism |  |  |  |  |  |
| 25. | Nightmares |  |  |  |  |  |
| 26. | Restless leg syndrome |  |  |  |  |  |

Daytime tiredness and sleepiness

|  | Answer the following questions based on your sleep behavior in the previous week | **Never** | **Less than once a week** | **1 – 2 nights a week** | **3 – 4 nights a week** | **Nightly/ Daily** |
| --- | --- | --- | --- | --- | --- | --- |
| 26. | Feeling tired in the morning |  |  |  |  |  |
| 27. | Daytime sleepiness |  |  |  |  |  |
| 28. | Daytime sleepiness during the lectures |  |  |  |  |  |
| 29. | Daytime sleepiness during free time |  |  |  |  |  |
| 30. | Daytime naps |  |  |  |  |  |

Miscellaneous questions

|  | **Variable** | **Excellent** | **Good** | **Satisfactory** | **Poor** |
| --- | --- | --- | --- | --- | --- |
| 31. | sleep Quality |  |  |  |  |
| 32. | Sleep quality on the night before an exam |  |  |  |  |
| 33. | Academic performance |  |  |  |  |
| 34. | Leisure time |  |  |  |  |
| 35. | Living conditions |  |  |  |  |
